# Retroauricular approach for carotid body tumor resection: a case report

**DOI:** 10.3389/fonc.2026.1918905

**Published:** 2026-10-01

**Authors:** Yi-Chan Lee, Wen-Yu Wu, Yao-Chang Wang

**Affiliations:** 1 Department of Otolaryngology - Head and Neck Surgery, Chang Gung Memorial Hospital, Keelung, Taiwan; 2 College of Medicine, Chang Gung University, Taoyuan, Taiwan; 3 Department of Medical Education, Chang Gung Memorial Hospital at Linkou, Taoyuan, Taiwan; 4 Department of Cardiovascular Surgery, Chang Gung Memorial Hospital, Keelung, Taiwan

**Keywords:** carotid body tumor, head and neck, quality of life, retroauricular, surgery

## Abstract

**Introduction:**

Carotid body tumors (CBTs) are rare and highly vascular paragangliomas arising at the carotid bifurcation. Surgical resection remains the standard treatment for CBTs. However, the conventional transcervical approach inevitably leaves a visible cervical scar, which may negatively affect quality of life in patients with cosmetic concerns.

**Case presentation:**

A 27-year-old woman presented with a progressively enlarging right neck mass over a 6-month period. Ultrasonography demonstrated a hypervascular lesion surrounding the carotid arteries, and fine-needle aspiration was avoided because of the vascular nature of the tumor. Contrast-enhanced computed tomography revealed a 4-cm enhancing mass at the carotid bifurcation, highly suggestive of a CBT. Because the patient expressed concern regarding a visible cervical scar, a retroauricular approach was selected after thorough discussion of its potential risks and benefits. The operation was performed by a head and neck surgeon with intraoperative assistance from a vascular surgeon. Complete tumor resection was successfully achieved despite the need for repair of two small arterial defects encountered during tumor mobilization. The operative time was 300 minutes, and the estimated blood loss was 900 mL. The postoperative course was uneventful, and no major complications or tumor recurrence were observed during 12 months of follow-up. The patient reported satisfaction with the cosmetic outcome, although no objective cosmetic assessment was performed.

**Conclusion:**

In carefully selected patients with Shamblin type I or II CBT, the retroauricular approach may represent a feasible hidden-scar alternative to the conventional transcervical approach.

## Introduction

Head and neck tumors comprise a heterogeneous group of benign and malignant neoplasms, many of which require surgical management (1–9). Carotid body tumors (CBTs), also known as carotid body paragangliomas, are rare and highly vascular neoplasms arising from paraganglionic chemoreceptor cells located at the carotid bifurcation (10–13). Because of their intimate anatomical relationship with the carotid arteries and adjacent cranial nerves, surgical resection of CBTs is considered technically challenging. Conventional transcervical access is generally the standard surgical approach for CBT resection. However, this technique inevitably results in a visible cervical scar. For patients with significant cosmetic concerns, the conventional approach may lead to decreased quality of life and psychological health (14). Hidden-scar techniques, such as the retroauricular and modified facelift approaches, have been increasingly applied in head and neck surgery with favorable cosmetic outcomes. Their feasibility has been demonstrated in a variety of head and neck procedures, including neck dissection, branchial cleft cyst surgery, and salivary gland surgery (15–20). Accordingly, the retroauricular approach may represent a feasible option for selected CBTs while avoiding a visible cervical scar.

In the present report, we describe the successful resection of a CBT through a retroauricular approach. To the best of our knowledge, this is the first English-language report of CBT resection through a retroauricular approach.

## Case description

A 27-year-old woman presented with a progressively enlarging right neck mass over six months. She had no relevant past medical history, family history, or systemic symptoms. Physical examination revealed a rubbery, non-tender mass measuring approximately 4 cm in the right upper neck. The lesion was located just inferior to the angle of the mandible and demonstrated obvious pulsation synchronous with the heartbeat. No overlying skin changes were noted (**Figure 1A**).

**Figure 1:**
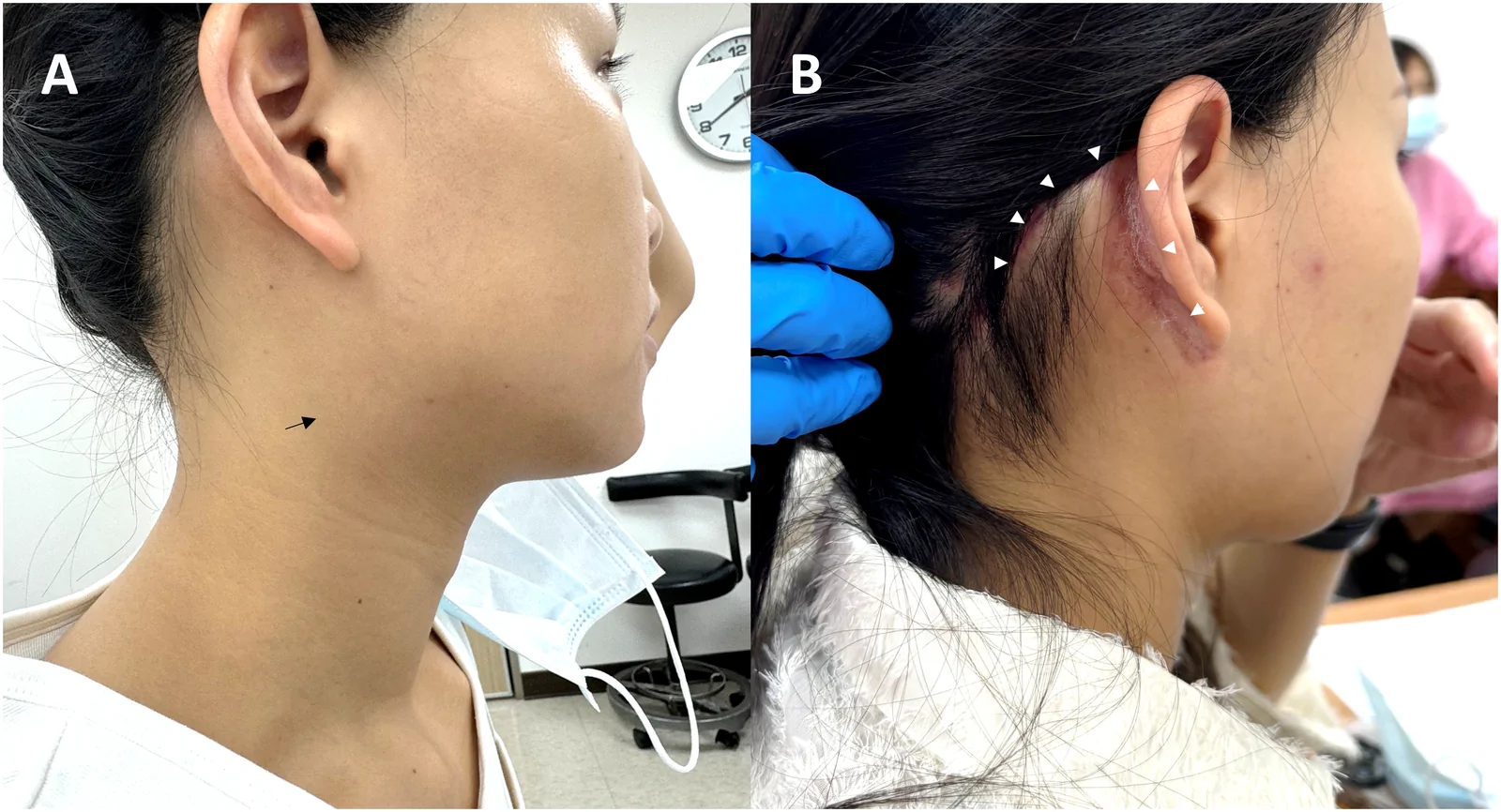
Appearance of the patient’s neck. **(A)** Preoperative appearance. The arrow indicates the carotid body tumor. **(B)** Appearance 3 months after surgery. The white arrowheads indicate the operative incision and scar.

Subsequent imaging studies were performed for further evaluation. Ultrasonography demonstrated a hypervascular lesion encasing the carotid arteries (**Supplementary Figure 1**). Given the highly vascular nature of the lesion and its close relationship to the carotid vessels, fine-needle aspiration cytology was not performed. Contrast-enhanced computed tomography revealed a 4-cm avidly enhancing mass with central necrosis at the carotid bifurcation, demonstrating the characteristic Lyre sign and highly suggestive of a CBT. According to the Shamblin classification, the tumor completely encased the external carotid artery (ECA), while part of the posterior surface of the internal carotid artery (ICA) remained free of tumor involvement. The lesion was therefore classified as a Shamblin type II CBT (**Figure 2A**) (10, 21). Magnetic resonance imaging and digital subtraction angiography were not performed because contrast-enhanced computed tomography provided sufficient anatomical information for diagnosis and surgical planning. Preoperative embolization was not performed because its routine use remains controversial, particularly for Shamblin type II CBT (22).

**Figure 2:**
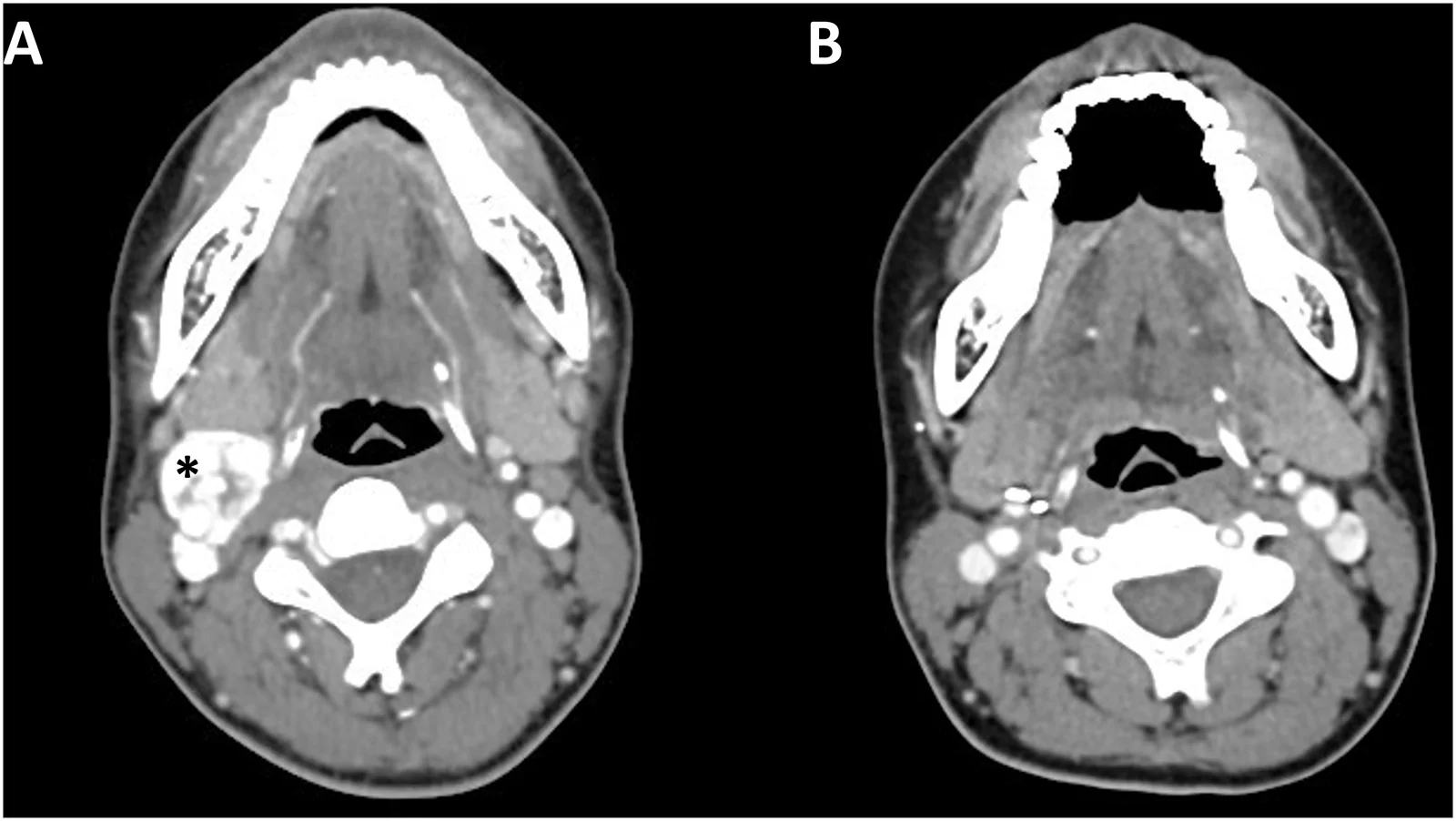
Computed tomography findings. **(A)** Preoperative contrast-enhanced computed tomography. The asterisk indicates the carotid body tumor. **(B)** Contrast-enhanced computed tomography at 10 months after surgery, showing no evidence of tumor recurrence.

Given the patient’s young age and the potential for progressive tumor growth, surgical resection was considered an appropriate treatment option. Although the conventional transcervical approach remains the standard surgical technique for CBT resection, it inevitably results in a visible cervical scar. The patient expressed significant concern regarding postoperative cosmetic outcomes because of her age and occupational requirements. After thorough discussion of the potential risks and benefits, a retroauricular approach was selected. The patient was also informed that conversion to a conventional transcervical approach would be performed if adequate surgical exposure or vascular control could not be safely achieved intraoperatively.

To maximize surgical safety, a multidisciplinary surgical approach involving both a head and neck surgeon and a vascular surgeon was utilized for tumor removal. Intraoperative nerve monitoring was utilized to facilitate preservation of the marginal mandibular branch of the facial nerve. Hemostatic dressing (QuikClot^®^) was available for hemorrhage control, and a rigid endoscope was used when necessary to assist visualization in areas with limited exposure.

The procedure was initiated by the head and neck surgeon through a retroauricular approach (**Supplementary Figure 2**). The incision was made behind the earlobe, following the retroauricular sulcus and extending posteroinferiorly within the occipital hairline. Skin flap elevation was performed in the supraperiosteal plane over the mastoid and then transitioned into the subplatysmal plane within the cervical region to achieve adequate surgical field exposure. After complete flap elevation, a Bookwalter self-retaining retractor system was assembled and positioned to maximize surgical exposure.

An incision was then made along the anterior border of the sternocleidomastoid muscle. The lateral aspect of the CBT was carefully dissected from the muscle, followed by meticulous blunt and sharp dissection of the medial aspect from the surrounding tissues (**Figure 3A**). Important neural structures, including the marginal mandibular branch of the facial nerve, hypoglossal nerve, vagus nerve, spinal accessory nerve, and ansa cervicalis, were identified and carefully preserved throughout the procedure.

**Figure 3:**
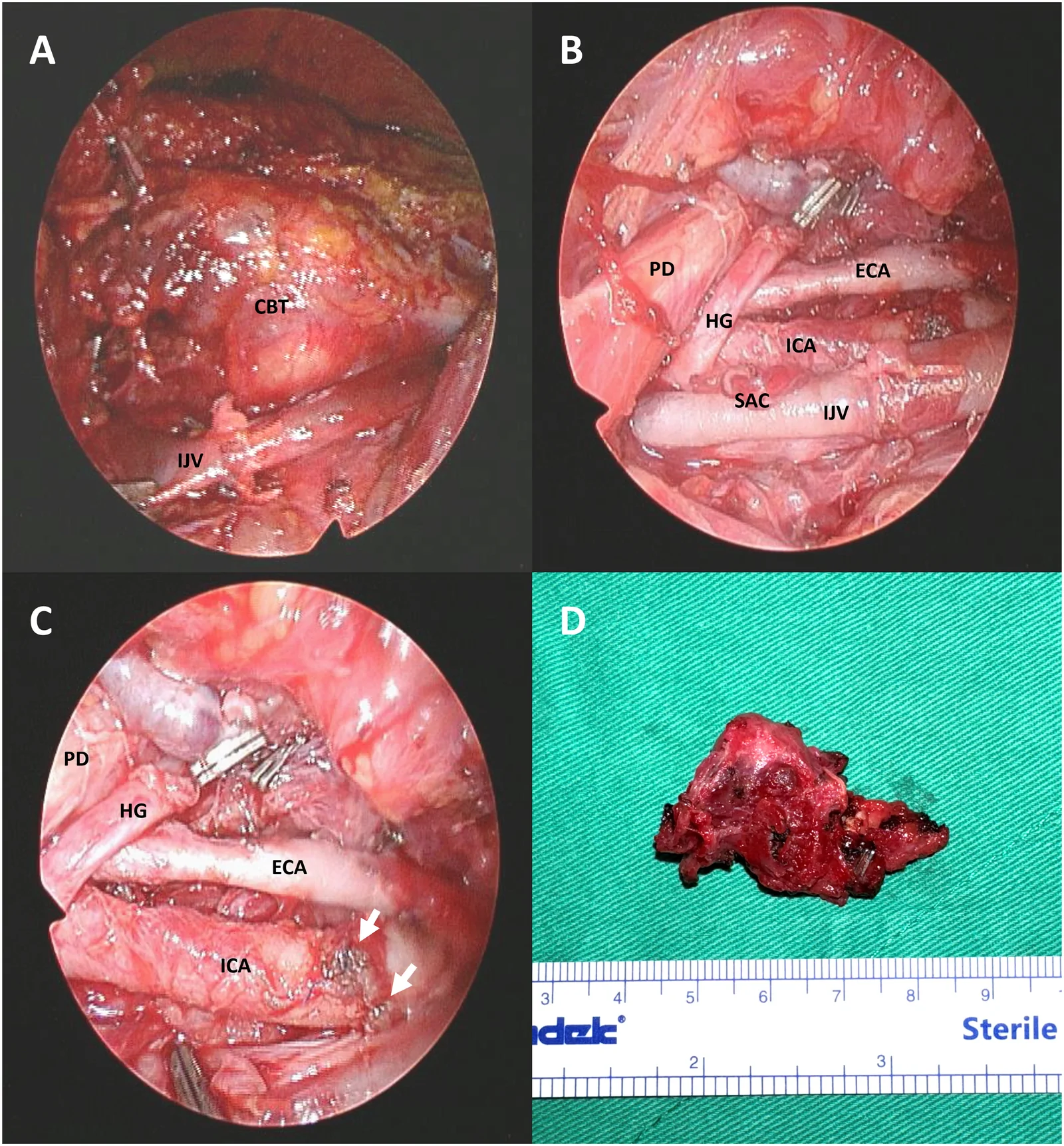
Intraoperative findings. **(A)** The carotid body tumor (asterisk) during initial dissection. **(B)** The operative field following en bloc tumor excision. **(C)** The carotid arteries after tumor excision. White arrowheads indicate the sites of arterial suture repair. **(D)** Gross specimen of the resected carotid body tumor. PD, posterior belly of the digastric muscle; HG, hypoglossal nerve; SAC, superior root of the ansa cervicalis; ICA, internal carotid artery; ECA, external carotid artery; CBT, carotid body tumor; IJV, internal jugular vein.

Proximal and distal vascular control was established using vessel loops placed around the common carotid artery, ICA and ECA. After confirming the extent of the tumor, dissection was initiated along the ICA using a meticulous periadventitial technique. During mobilization of the tumor from the ICA, two small arterial defects were encountered, at which point the vascular surgeon took over the operative field and immediately repaired the defects. The vascular surgeon subsequently completed the dissection of the tumor from the ICA. Multiple feeding branches from the ECA were sequentially identified and ligated by the head and neck surgeon. Once the entire CBT was fully mobilized, it was resected in an en bloc fashion (**Figures 3B–D**). The surgical field was irrigated and the wound was closed in layers. A Jackson-Pratt drain was placed to reduce the risk of postoperative hematoma and seroma formation. The total operative time was 300 minutes, and the estimated blood loss was 900 mL.

Postoperative recovery was uneventful, and no major complications occurred. The patient was discharged on postoperative day 4. Histopathological examination confirmed the diagnosis of paraganglioma. The patient experienced mild postoperative shoulder tightness, which resolved completely within four months. Contrast-enhanced computed tomography performed 10 months after surgery demonstrated no evidence of tumor recurrence (**Figure 2B**). Additional preoperative and postoperative contrast-enhanced computed tomography images are shown in **Supplementary Figures 3** and **S4**. From a cosmetic perspective, the surgical scar was effectively concealed within the retroauricular sulcus and hairline and was difficult to identify even on close inspection. The patient reported satisfaction with the cosmetic outcome and no adverse impact on quality of life (**Figure 1B**). She remains under regular follow-up and the clinical timeline and interventions are summarized in **Figure 4**.

**Figure 4:**
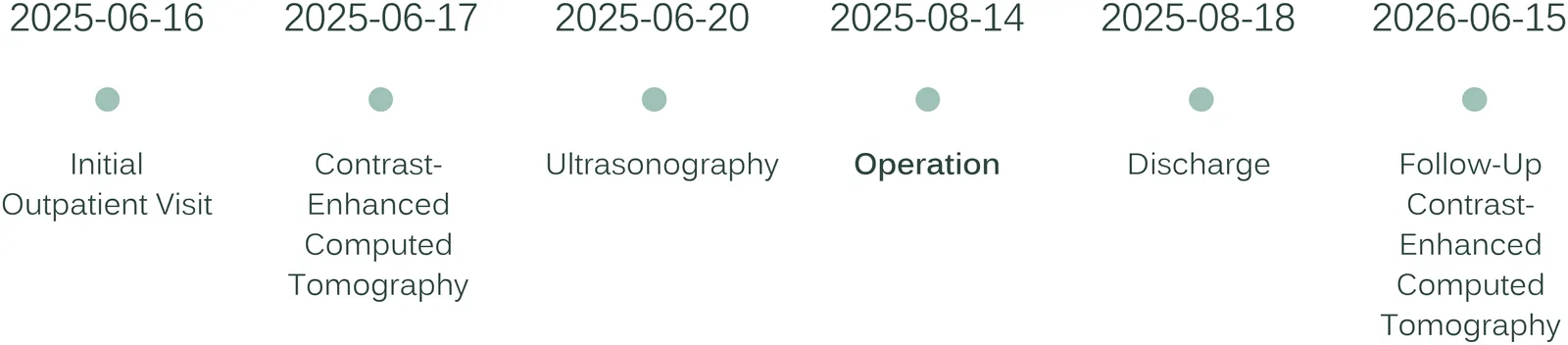
Timeline of clinical events and interventions.

## Discussion

To our knowledge, this is the first report in the English literature describing CBT resection through a retroauricular approach. The present case demonstrates that this hidden-scar technique can be applied to CBT surgery while maintaining adequate exposure for cranial nerve identification, vascular control, and complete tumor resection. In addition to its technical feasibility, the approach provided a favorable cosmetic outcome by concealing the scar within the retroauricular sulcus and hairline. Previous studies have demonstrated the feasibility of retroauricular approaches in a variety of head and neck procedures (15–20). Our surgical team has accumulated sufficient experience with this technique over the past decade, thereby enabling us to extend its application to CBT resection.

In the present case, all important neural structures were successfully identified and preserved. Adequate proximal and distal vascular control of the ICA and ECA was also achieved, suggesting that the retroauricular approach can provide adequate exposure for CBT dissection in carefully selected patients. Nevertheless, meticulous dissection remains essential to successful surgery. Instruments such as endoscopes, energy devices, and robotic systems may also be used to facilitate surgical procedures. Although robotic assistance was not utilized in the current case, endoscopic assistance was employed to inspect and confirm the extent of dissection. Despite the need for repair of two small arterial defects encountered during tumor mobilization, complete CBT resection was achieved without major postoperative complications. Compared with previously reported outcomes of conventional resection for similar CBTs, the operative time in the present case was generally comparable, whereas intraoperative blood loss was higher. This was likely related to the repair of the arterial defects encountered during tumor mobilization, highlighting the importance of meticulous vascular control and multidisciplinary collaboration (23, 24).

Several considerations should be taken into account before applying this approach. First, the differential diagnosis of a carotid bifurcation mass includes vagal and cervical sympathetic chain schwannomas. Characteristic patterns of carotid vessel displacement on imaging, including carotid bifurcation splaying in CBT, usually allow differentiation and facilitate accurate preoperative diagnosis and surgical planning (25, 26). Second, surgeons should possess substantial experience with retroauricular techniques and be prepared to convert to a conventional transcervical approach when necessary. Because CBTs are highly vascular lesions intimately associated with major vessels, multidisciplinary collaboration with vascular surgeons may further enhance surgical safety. Such vascular injuries are not uncommon even during conventional transcervical resection and further highlight the value of multidisciplinary collaboration (27). Appropriate patient selection is also crucial for the safe application of this technique. In the present case, the tumor was classified as Shamblin type II, with part of the posterior surface of the ICA remaining free of tumor encasement (10, 21). In contrast, Shamblin type III tumors are technically more challenging because of more extensive ICA encasement and often require substantially wider surgical exposure, with vascular reconstruction occasionally necessary. Therefore, this technique should not be considered a replacement for the conventional transcervical approach but rather an alternative option for carefully selected patients with Shamblin type I or II tumors, particularly those with favorable anatomy and significant cosmetic concerns. Finally, although the patient was satisfied with the cosmetic outcome and no permanent cranial nerve deficits or major vascular complications occurred, this study remains a single case report, and cosmetic outcomes were not objectively assessed using a validated scar assessment tool. Additional cases and longer follow-up are needed to further evaluate the long-term safety, reproducibility, and broader applicability of this technique.

## Conclusion

To our knowledge, this is the first reported case of CBT resection performed through a retroauricular approach. In carefully selected patients with Shamblin type I or II CBT, favorable anatomy with limited carotid artery encasement, and significant cosmetic concerns, this hidden-scar technique may be considered an alternative to the conventional transcervical approach.

## Data Availability

The raw data supporting the conclusions of this article will be made available by the authors, without undue reservation.
