# Supplementary material for: Retroauricular approach for carotid body tumor resection: a case report
Source: Front Oncol. 2026 Oct 1;16:1918905. doi: 10.3389/fonc.2026.1918905 (PMC13668566; doi:10.3389/fonc.2026.1918905)
Supplement: Supplementary file 1 [file DataSheet1.docx]

**Figure S1. Ultrasonographic findings of the carotid body tumor.**

(A) Gray-scale ultrasonography of the carotid body tumor.
(B) Color Doppler ultrasonography demonstrating the vascularity of the carotid body tumor.
Arrow, internal carotid artery; arrowhead, branches of the external carotid artery.

**Figure S2. Schematic diagram of the retroauricular approach.**

The red line indicates the design of the retroauricular incision**.**

**Figure S3. Coronal and sagittal contrast-enhanced computed tomography images before and after surgery.**

(A) Coronal contrast-enhanced computed tomography image before surgery.
(B) Coronal contrast-enhanced computed tomography image after surgery.
(C) Sagittal contrast-enhanced computed tomography image before surgery.
(D) Sagittal contrast-enhanced computed tomography image after surgery.
Asterisk, carotid body tumor.

**Figure S4. Relationship between the carotid body tumor and the carotid vessels on preoperative contrast-enhanced computed tomography.**
The carotid body tumor was located at the carotid bifurcation. (A) Sagittal view. (B) Axial view.
Arrow, external carotid artery; arrowhead, internal carotid artery; asterisk, internal jugular vein.
